# Heat Shock Proteins in Urine as Cancer Biomarkers

**DOI:** 10.3389/fmed.2021.743476

**Published:** 2021-10-08

**Authors:** Zarema Albakova, Diogo Dubart Norinho, Yana Mangasarova, Alexander Sapozhnikov

**Affiliations:** ^1^Department of Biology, Lomonosov Moscow State University, Moscow, Russia; ^2^Data Science Department, NOS SGPS, Porto, Portugal; ^3^National Research Center for Hematology, Moscow, Russia; ^4^Department of Immunology, Shemyakin and Ovchinnikov Institute of Bioorganic Chemistry of Russian Academy of Sciences, Moscow, Russia

**Keywords:** heat shock proteins, biomarkers, cancer, urine, machine learning

## Abstract

Heat shock proteins (HSPs) are a large family of molecular chaperones, which have shown to be implicated in various hallmarks of cancer such as resistance to apoptosis, invasion, angiogenesis, induction of immune tolerance, and metastasis. Several studies reported aberrant expression of HSPs in liquid biopsies of cancer patients and this has opened new perspectives on the use of HSPs as biomarkers of cancer. However, no specific diagnostic, predictive, or prognostic HSP chaperone-based urine biomarker has been yet discovered. On the other hand, divergent expression of HSPs has also been observed in other pathologies, including neurodegenerative and cardiovascular diseases, suggesting that new approaches should be employed for the discovery of cancer-specific HSP biomarkers. In this study, we propose a new strategy in identifying cancer-specific HSP-based biomarkers, where HSP networks in urine can be used to predict cancer. By analyzing HSPs present in urine, we could predict cancer with approximately 90% precision by machine learning approach. We aim to show that coupling the machine learning approach and the understanding of how HSPs operate, including their functional cycles, collaboration with and within networks, is effective in defining patients with cancer, which may provide the basis for future discoveries of novel HSP-based biomarkers of cancer.

## Introduction

Heat shock proteins (HSPs) are molecular chaperones that are classified into families such as HSP70, HSP90, HSP40, HSPB, HSP110, and chaperonins (1). Members of HSP families are located in different cellular compartments such as cytosol, nucleus, lysosome, endoplasmic reticulum, and mitochondria (1–3). Several studies reported high levels of HSP70, HSP90, HSP40, HSPB, and chaperonins in plasma, serum, and plasma-/urine-derived exosomes of the patients in different types of cancer compared to healthy individuals (3–15). This has opened new perspectives on the use of HSPs as biomarkers of cancer. However, abnormal expression of HSPs has also been observed in several other pathologies including cardiovascular and neurodegenerative diseases (16–18). For example, Li and his colleagues showed that high expression of HSP70 in plasma positively correlated with heart failure (19). Therefore, new strategies should be used for the identification of cancer-specific HSP biomarkers. Since HSPs are tightly linked to the stress response, level of individual HSP members in the clinical samples may not be enough for precise prediction of cancer. Herein, we used a machine learning approach for the identification of HSP-based urine biomarkers of cancer. We show that coupling machine learning approach and the understanding of how HSPs operate in networks may be effective in diagnosing cancer. To the best of our knowledge, this is the first study that explores HSP secreted in urine for prediction of cancer and the primary study to assess the relationships between different HSP networks and cochaperones for the discovery of clinically useful HSP-based biomarkers of cancer.

## Methods

We used publicly available mass spectrometry dataset that contains samples from 231 donors (20). Urine samples were derived from the patients with gastric cancer (GC) (*n* = 47), esophageal cancer (EC) (*n* = 14), lung cancer (LC) (*n* = 33), bladder cancer (BC) (*n* = 17), cervical cancer (CCA) (*n* = 25), colorectal cancer (CRC) (*n* = 22), and benign lung diseases (LDs) such as chronic obstructive pulmonary disease (COPD) (*n* = 17) and pneumonia (PM) (*n* = 23) as well as from the healthy volunteers (Control, CTL) (*n* = 33) (20). Urine samples were centrifuged at 200,000 g for 70 min and absolute protein amounts were measured by liquid chromatography with tandem mass spectrometry (LC-MS/MS) and presented as intensity-based fraction of total (iFOT; displayed in 10^5^) representing normalized intensity for each protein (20). HSPs such as HSP70, HSP90, HSP40, HSP27, HSP110, chaperonins, and cochaperones were included in the analysis (Supplementary Table 1). Proteins that have > 30% of 0.0099 (missing values) were excluded from the analysis.

The expression level for each protein was measured for CTL and six groups of cancers (LC, BC, CCA, CRC, EC, and GC). Since the data were not normally distributed, nonparametric tests were used. The procedure was divided into two stages such as the Kruskal–Wallis (KW) test for all the proteins followed by a *post-hoc* Dunn's test using CTL as reference (21). Bonferroni multiple comparison test (MCT) correction in its multistep variant, known as Holm–Bonferroni correction, was also used (22).

The cancer prediction model was trained on HSP and their cochaperones to isolate their effects in cancer prediction. Taking into account that HSPs are located in different cellular compartments as well as exist in different forms (constitutive/stress-inducible) and require cochaperones for their functional cycles, while also working in networks, we introduced into the model various combinations of simple ratios and multiplication strategies. For example, to isolate the effect of HSP90 homologs, we used the relationship between the level of cytosolic HSP90 homolog to the level of mitochondrial HSP90 homolog in a simple ratio of HSP90AA1/TRAP1, constitutive HSP90 isoform to stress-inducible HSP90 in a simple ratio of HSP90AB1/HSP90AA1, cochaperone level to the HSP90α level in a simple ratio of FKBP4/HSP90AA1, etc. (Supplementary Table 2). As a result, a cancer prediction model was created using XGBoost with a tree booster. A binary classification model was built to discriminate the cancer patients (LC, BC, CCA, CRC, EC, and GC) from the non-cancer group (LD and CTL). The performance of the method was evaluated through 10-fold stratified cross-validation. By splitting the data into 10-fold, iteratively training in 9-fold and testing on the remaining fold, we mimic the effect of 10 distinct datasets. This enables us to estimate the generalization error of our model and prevent overfitting, therefore ensuring that the model would generalize well to new data. Bayesian optimization was used to tune hyperparameters. We computed features importance using the gain metric, which measures the loss reduction of adding a split with that feature. Let ξ_*l*_ be the set of features at the *l*^*th*^ step tuning:

1. Start the first iteration with all the features (ξ_1_).
  - Initialize the Bayesian optimization:
    - Randomly, select *n*_1_ points {ϕ_1_, …, ϕ_*n*_1__} located within user defined boundaries:
      1. Train with hyperparameter set ϕ_*i*_ and evaluate the model using K-fold cross-validation with log-loss.
  - Perform the Bayesian optimization:
    - Sequentially, select *n*_2_ points:
      1. ϕ_*j*_ is the point that maximizes the upper confidence bound of the posterior distribution of the Gaussian process by given the data points {ϕ_1_, …, ϕ_*j* − 1_} for *j* > *n*_1_.
  - Of the *n*_1_ + *n*_2_ combinations tried, select the set of hyperparameters that minimize the log-loss such that Θ_1_ = *argmin*_{_ϕ__1_, …, ϕ_*n*_1_+*n*_2__}_ *log loss*.
  - For each of the *K* models with parameters Θ_1_ trained in the K-fold cross-validation, extract the feature importance and then compute the average for each feature.
  - Remove all the features whose importance is equal to the minimum.
2. For iteration *l*:
  - Initialize the Bayesian optimization and randomly select *n*_1_ new points.
  - Probe all {Θ_1_, …, Θ_*l* − 1_} the points.
  - Perform the Bayesian optimization by sequentially selecting *n*_2_ points.
  - Select Θ_*l*_ = *argmin*_{_ϕ__1_, …, ϕ_*n*_1_+*n*_2_+*l* − 1_}_
  - Perform feature selection
  - Stop if there is only one feature left or all the features have the same importance, otherwise, continue
3. Stop when reach zero feature.
4. Select ξ_*k*_, Θ_*k*_ corresponding to the minimum log loss across all the iterations.

## Results

Heat shock proteins and cochaperones including HSP90AB1, TRAP1, FKBP4, HSPA9, HSPB5, CCT1, and CCT5 were identified as differentially expressed proteins (Table 1). CCT1, CCT5, and FKBP4 showed significantly lower expression in the cancer patients compared to the healthy volunteers, whereas HSPA9 and TRAP1 showed a significantly higher expression in patients with cancer compared to the control group for the most cancer types. HSPB5 showed significantly higher expression only in the CCA patients compared to the healthy volunteers (Table 1). HSP90AB1 showed a significantly lower expression in the patients with GC and CRC compared to CTL (Table 1).

**Table 1:** Differentially expressed HSPs and cochaperones in the urine of the cancer patients compared to healthy volunteers by Dunn's test with Holm–Bonferroni correction.

**Cancer type**	**CCT1**		**CCT5**		**FKBP4**		**HSPB5**		**HSP90AB1**		**HSPA9**		**TRAP1**	
	**Test statistic**	***p*** **-value**	**Test statistic**	***p*** **-value**	**Test statistic**	***p*** **-value**	**Test statistic**	***p*** **-value**	**Test statistic**	***p*** **-value**	**Test statistic**	***p*** **-value**	**Test statistic**	***p*** **-value**
**LC**	−3.6	1.69E-03	−4.1	2.41E-04	−5.0	4.04E-06	0.25	1.00	−2.2	0.119	4.3	1.02E-04	3.5	2.28E-03
**BC**	−1.9	0.125	−2.9	1.02E-02	−4.1	1.36E-04	−0.56	1.00	0.61	1.00	4.3	8.80E-05	3.5	2.76E-03
**CCA**	−3.9	5.92E-04	−3.4	2.37E-03	−2.7	1.19E-02	2.7	4.07E-02	−0.68	1.00	1.9	7.84E-02	2.8	1.70E-02
**CRC**	−3.4	2.47E-03	−3.8	6.56E-04	−4.3	8.05E-05	−1.9	0.278	−2.7	3.77E-02	2.1	7.84E-02	1.9	0.119
**EC**	−2.8	1.53E-02	−2.3	4.77E-02	−0.84	0.402	−1.1	1.00	0.58	1.00	3.1	7.18E-03	3.3	3.69E-03
**GC**	−1.4	0.164	−1.2	0.216	−3.4	1.91E-03	0.76	1.00	−2.7	3.77E-02	2.2	7.27E-02	3.4E-02	0.973

*LC, lung cancer; BC, bladder cancer; CCA, cervical cancer; CRC, colorectal cancer; EC, esophageal cancer; GC, gastric cancer; HSPs, heat shock proteins*.

**Figure 1:**
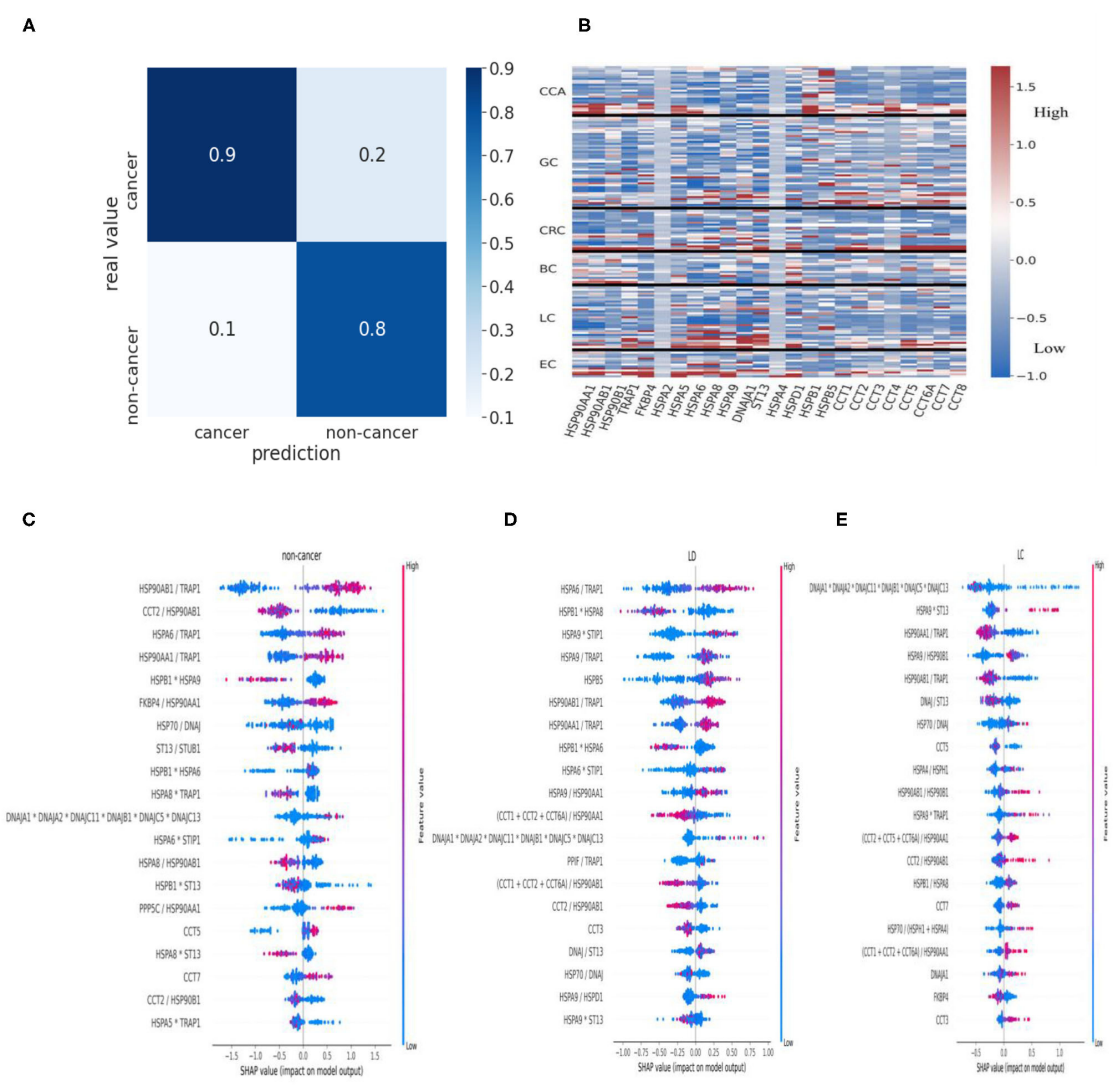
HSPs in urine as biomarkers of cancer. **(A)** Confusion matrix for the cancer prediction model. **(B)** Heatmap of z-score normalized HSP expression levels in the urine of the patients with different types of cancer. Values were clipped to the 1st percentile of the *z*-scores and to the 97th percentile to minimize the effect of outliers. **(C)** HSPs and cochaperones in cancer and non-cancer patients. Negative values indicate a positive contribution of specific proteins to the probability that a patient has cancer. Positive SHAP values indicate that the corresponding values of the proteins are associated with lower chances of the patient having cancer. For simplicity, we presented HSPA2+HSPA6+HSPA8+HSPA12+HSPA5 as “HSP70” and DNAJA1+DNAJA2+DNAJC11+DNAJB1+DNAJC5+DNAJC13 as “DNAJ”. **(D,E)** SHAP summary plots for the cancer prediction model. HSPs in urine were used to identify the critical proteins and the protein ratios in patients with benign lung disease (LD) such as PM and COPD **(D)** and LC patients **(E)**. HSPs, heat shock proteins; PM, pneumonia; COPD, chronic obstructive pulmonary disease; LC, lung cancer; SHAP, Shapely Addictive Explanations.

Remarkably, the cancer prediction model trained on HSPs and cochaperones resulted in 90% precision and a balanced accuracy of 84.61% (accuracy of 87.041%) averaged over the 10 cross-validation test folds (Figure 1A). In order to identify proteins, which positively contributed to the cancer prediction model, we have implemented the Shapely Addictive Explanations (SHAP) approach. Low levels of HSP90AB1/TRAP1, HSPA6/TRAP1, and HSP90AA1/TRAP1 in urine increase the probability of the patient having cancer, whereas low levels of CCT2/HSP90AB1 and HSPB1^*^HSPA9 in urine are strongly associated with non-cancer groups (Figure 1C). In order to assess the differences in the level of HSPs across different types of cancer, we constructed a heatmap, representing the z-score of HSPs for each patient (Figure 1B). HSP90AA1 and HSPD1 showed to be highly expressed in BC; HSPB1 and HSBP5 in CCA; ST13, DNAJA1, and HSPA8 in LC; FKBP4 and HSPA8 in EC (Figure 1B). HSPA2 and HSPA4 did not seem to be affected in different types of cancer (Figure 1B).

Higher levels of both constitutive and stress-inducible HSP90 isoforms in relationship to mitochondrial HSP90 isoform TRAP1 are associated with benign lung diseases such as PM and COPD, whereas a higher level of TRAP1 to HSP90AA1 and HSP90AB1 is associated with lung cancer (Figures 1D,E). In contrast to patients with PM, a low level of CCT5 and high levels of HSPA9^*^TRAP1 and CCTs/HSP90AA1 are associated with LC (Figure 1E; Supplementary Figure 1A). Furthermore, lower expression of HSP90AA1/TRAP1 and HSP90AB1/TRAP1 positively contributed to LC compared to higher expression of HSP90AA1/TRAP1 and HSP90AB1/TRAP1 in the COPD patients (Figure 1E; Supplementary Figure 1B). Overall, urine samples contain cancer-specific HSP signatures. Therefore, these HSP signatures may be used to distinguish cancer from noncancer patients and patients with benign disease as well as they may be further used to identify specific types of cancer; however, this requires further investigation.

## Discussion

Heat shock proteins are ubiquitously expressed as molecular chaperones, which support tumor growth and survival (23). Cells possess various families of HSPs with distinct functions, often working in collaboration to perform proper folding and degradation of client proteins (24, 25). Several studies reported altered expression of HSPs in malignant cells compared to their normal cell counterparts (3–15). Furthermore, overexpression of HSPs has been linked with tumor aggressiveness, metastasis, and poor prognosis (2, 24, 26–29). In this study, we aimed at exploring the potential of HSPs in urine as biomarkers of cancer. We showed that HSP chaperone networks can be used to predict cancer with ~90% precision in 10-fold cross-validation. We highlighted that understanding of HSP chaperone system and the notion of how HSPs operate are critical for prediction of cancer.

Our approach started with an identification of differentially expressed HSP proteins in different types of cancer compared to healthy volunteers. We showed that different HSP members are up- and down-regulated in different types of cancer, suggesting that a specific type of cancer has distinct HSP signatures (Table 1). We then developed a cancer prediction model, which reflected the way how HSP chaperone networks work. The model is based on the notion that HSP networks work in collaboration with each other as well as with cochaperones and that there also may be some shift in the proportion of different HSP homologs in the cancer patients compared to the healthy individuals and the benign patients, leading to all of these changes being captured by machine learning approach. Using this approach, we could predict cancer with 90% precision (Figure 1A). Furthermore, our cancer prediction model could discriminate between various types of cancer based on the expression of distinct HSPs in urine samples, which may help in diagnosing specific subtypes of cancer among a heterogeneous group of tumors, such as lymphoma or breast cancer. In this regard, Klimczak et al. (30) used The Cancer Genome Atlas and KM plotter databases to show that expression of six HSPs including *HSPA2, DNAJC20, HSP90AA1, CCT1, CCT2*, and *CCT6A* can be used to predict prognosis in patients with breast cancer (30). Furthermore, upregulation of distinct HSPs was associated with either estrogen receptor-positive, progesterone receptor-positive, or human epidermal growth factor receptor 2-positive breast cancers (30). Therefore, the identification of type-specific HSP signatures in a heterogeneous group of tumors warrants further investigation.

It is also interesting to see the changes in HSPs between patients with benign lung disease and lung cancer patients (Figures 1D,E). Patients with lung disease have a higher level of cytoplasmic HSP90 homologs (HSP90AA1 and HSP90AB1) in relationship to mitochondrial HSP90 homolog (TRAP1), whereas patients with lung cancer have a higher level of TRAP1 to the level of cytoplasmic HSP90 (Figures 1D,E). Furthermore, the level of HSP70 to its cochaperone DNAJ/HSP40 does not seem to change between benign lung disease and cancer in contrast with a higher level of ST13 to DNAJ associated with lung cancer (Figures 1D,E). During the HSP70 functional cycle, ST13, also known as Hsc70-interacting protein (Hip), preferentially binds to the ADP-bound state of HSP70–peptide complexes, slowing the release of ADP from HSP70-nucleotide binding domain, thus, promoting degradation of HSP70 clients (24, 31, 32). This may suggest that HSP70 is predominantly “freezed” in its high-affinity ADP state in lung cancer patients and that the role of Hip should be further investigated in the context of cancer. The levels of CCTs also seem to influence the shift from lung disease to lung cancer (Figures 1D,E; Supplementary Figure 1). This provides a good example of the specific HSPs that made a positive contribution to shifting a balance from the benign disease state to cancer. Further understanding of HSP changes between benign disease and cancer may potentially provide clues for the discoveries of novel HSP-based biomarkers and therapeutic targets.

In conclusion, coupling the machine learning approach and understanding of how HSPs operate, including their functional cycles as well as collaboration with and within networks, are certainly effective in identifying specific types of cancer, which may form the basis for future discoveries of novel HSP-based biomarkers of cancer.

## Conclusion

Heat shock proteins are molecular chaperones that are aberrantly expressed in cancer patients and shown to be implicated in the various stages of cancer development. We hypothesized that HSPs in urine can be used to predict cancer. We show that HSPs can be used to identify cancer patients with nearly 90% precision based on HSP signatures in urine. We highlighted that understanding of HSP networks and how HSP operates in cells are crucial for the identification of HSP-based biomarkers of cancer. Further understanding of the HSP chaperone system may help in the development of effective type-specific biomarkers of cancer.

## Data Availability Statement

The original contributions presented in the study are included in the article/Supplementary Material, further inquiries can be directed to the corresponding author/s.

## Author Contributions

ZA collected the resources, contributed to the conceptualization, writing, review and editing of the manuscript, formal analysis and finance acquisition. DDN contributed to methodology, machine learning, review and editing of the manuscript. YM and AS provided administrational support. All the authors have read and agreed to the published version of the manuscript.

## Funding

This research was funded by the RFBR, project number 20-315-90081.

## Conflict of Interest

The authors declare that the research was conducted in the absence of any commercial or financial relationships that could be construed as a potential conflict of interest.

## Publisher's Note

All claims expressed in this article are solely those of the authors and do not necessarily represent those of their affiliated organizations, or those of the publisher, the editors and the reviewers. Any product that may be evaluated in this article, or claim that may be made by its manufacturer, is not guaranteed or endorsed by the publisher.
