# Supplementary material for: Heat Shock Proteins in Urine as Cancer Biomarkers
Source: Front Med (Lausanne). 2021 Oct 8;8:743476. doi: 10.3389/fmed.2021.743476 (PMC8531591; doi:10.3389/fmed.2021.743476)
Supplement: Supplementary file 1 [file Data_Sheet_1.docx]

Supplementary Material

A B


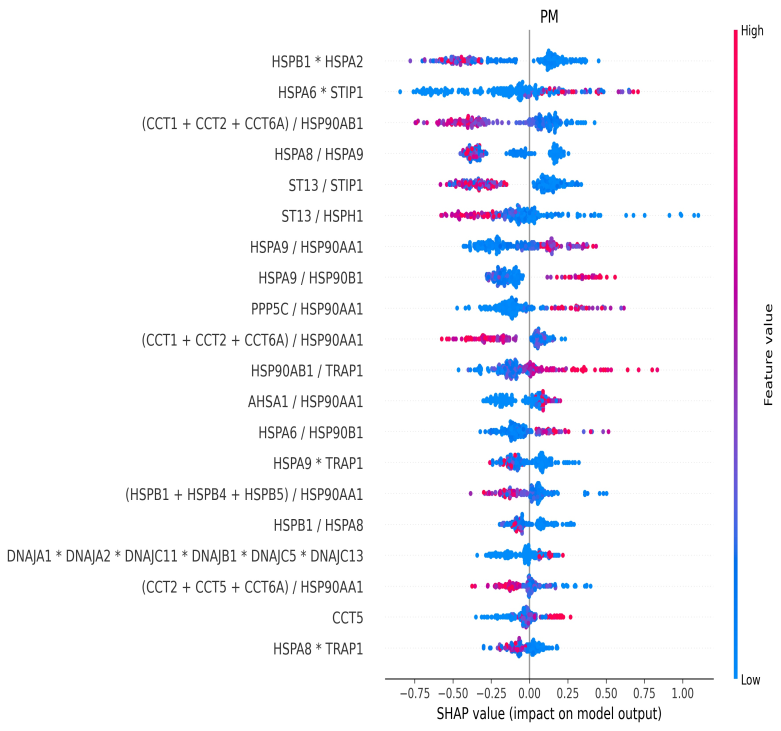

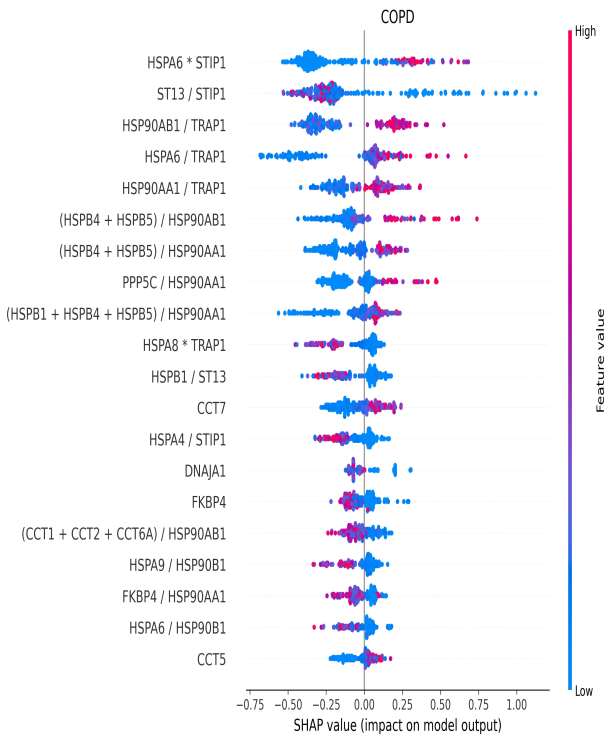


**Supplementary Figure 1.** HSPs in urine samples of patients with benign lung disease. SHAP summary plots for identification of critical protein rations in patients with PM (A) and COPD (B). PM, pneumonia; COPD, chronic obstructive pulmonary disease.
